# Head and neck lipoblastomas: Report of 3 cases and review of the literature

**DOI:** 10.1016/j.ijscr.2021.106050

**Published:** 2021-06-04

**Authors:** Boutaina Merzouqi, Mohammed Laachoubi, Youssef Oukessou, Mohammed Mahtar

**Affiliations:** a ENT Department, Face and Neck Surgery, Hospital August, 20'1953, University Hospital Center IBN ROCHD, Casablanca, Morocco; b Faculty of Medicine and Pharmacy, Hassan II University of Casablanca, B.P 5696, Casablanca, Morocco

**Keywords:** Lipoblastoma, Lipoblastomatosis, Adipose tumor, Cervical mass, Pediatric

## Abstract

**Introduction:**

Lipoblastoma is a rare benign tumor arising from embryonic white fat which occurs in the early childhood. It usually arises on the extremities and considered as a rare cause of a pediatric head and neck masses. The aim of this study is to shed light on lipoblastomas as a differential diagnosis of rapidly growing soft fatty masses of children in neck and head area.

**Patients and methods:**

A retrospective review of 3 patients with lipoblastoma, underwent Surgical resection (case 1 and 2) by cervical approach. The third patient with a facial lipoblastoma was not operated due to the high risk of facial paralysis. Review of literature, diagnostic methods and genetics of lipomatous tumors are discussed.

**Results:**

Complete surgical excision via a cervical approach demonstrated irregular lobules of immature fat cells separated by a loose, myxoid connective tissue. Histology analysis confirmed the diagnosis of lipoblastoma.

**Discussion:**

Lipoblastoma is a rare childhood tumor, even rarer in head and neck area. The pathogenesis is unknown, though it is believed to arise from altered embryogenesis of human white fat and genetic predisposition, as chromosome 8 abnormalities may be implicated in the development of lipoblastoma. The presumptive diagnosis is performed by imaging. The most important differential diagnosis of lipoblastoma is myxoid liposarcoma. The mainstay of treatment is complete non-mutilating resection of the tumor to avoid recurrence.

**Conclusion:**

Lipoblastoma should be suspected in case of heterogeneous fatty tumor in head and neck area, and included as a differential diagnosis of cervical masses in children younger than 3 years. The mainstay of treatment is complete surgical excision with a good prognosis.

## Highlights

• Lipoblastoma should be suspected in case of heterogeneous fatty tumor in head and neck area, in children younger than 3 years.
• Otolaryngologist should be familiar with this benign tumor when evaluating soft-tissue mass in the head and neck region.
• Complete non-mutilating resection of the tumor is the treatment of choice
• clinical follow-up of these lesions is necessary to avoid recurrence and malignant transformation.

## 1 Introduction

Lipoblastomas are very rare benign tumors arising from embryonic white fat which occurs in the early childhood. They are characterized by rapid growth, with symptoms depending on their location, and can cause deformity or compression of adjacent structures [1,2].

Lipoblastomas represent 4% to 6% of soft-tissue tumors in children, with a slight male predominance [3,4]. Usually, they arise on the extremities and considered as a rare cause of a pediatric head and neck masses [5].

There are two clinical forms with similar histology: lipoblastoma is a localized, superficial and encapsulated form, accounting for 70% of cases, and lipoblastomatosis is a diffuse form, infiltrating the adjacent muscle tissues [[1], [2], [3]].

Diagnosis is often suggested by clinical and radiological findings, especially magnetic resonance imaging (MRI) but can only be confirmed by histological examination which demonstrates well-defined adipocyte lobules, uniform growth, absent atypia, and lipoblasts [6].

Histologically there is an overlap between lipoblastoma and myxoid liposarcoma. Liposarcoma is a malignant tumor more common after 15 years of age, especially in the third decade of life [7].

The mainstay of treatment is complete non-mutilating resection of the tumor to avoid recurrence [8].

The aim of this study is to shed light on lipoblastomas as a differential diagnosis of rapidly growing soft fatty masses of children in head and neck area, and to emphasize the importance of complete resection while operating lipoblastoma cases.

## 2 Patients and methods

Here we present three clinical cases of head and neck lipoblastoma treated in the otolaryngology head and neck department of Casablanca university hospital. All surgeries were performed by a senior professor. Data was collected retrospectively. This work has been reported in line with the SCARE criteria [9].

### 2.1 Case 1

**Fig. 1:**
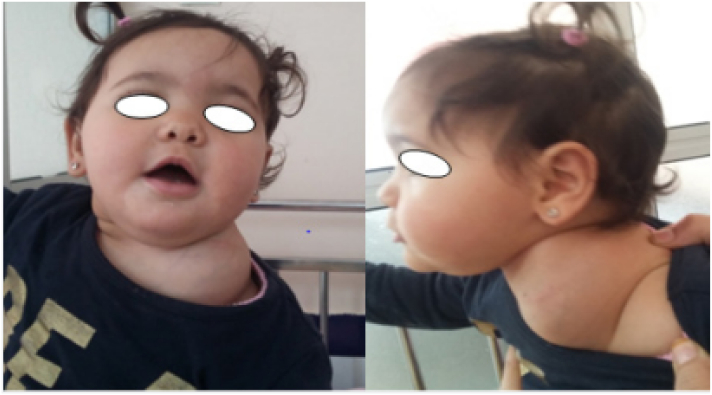
Pre-operative image of the large supraclavicular mass.

A 10-month-old girl, presented with a 6-month history of an enlarging, painless left side neck mass growing progressively over the last 2 months. There was no history of trauma or infection. On physical examination, there was a painless, non-pulsatile, firm left-sided cervical mass in the supraclavicular area measuring approximately 6 cm of large diameter, fixed to the underlying structures, mobile relative to the overlying skin (Fig. 1), with no inflammatory signs. No adenopathy or cranial nerves deficit was observed.

**Fig. 2:**
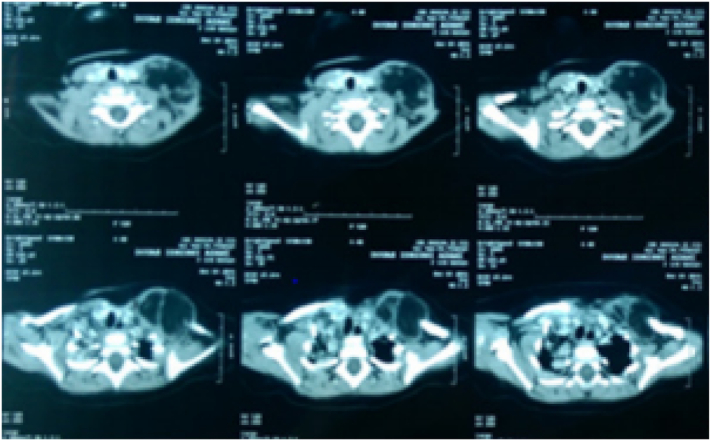
Axial neck CT scan section showing a well limited supraclavicular lobulated mass (62 × 43 × 41 mm) encroaching upon the sternocleidomastoid muscle.

Neck CT scan revealed a well limited supraclavicular lobulated mass, with a greasy density similar to that of the subcutaneous fat, crossed by internal septa, measuring 62 × 43 × 41 mm. The mass came into contact with the subclavian blood vessels which remain permeable (Fig. 2).

**Fig. 3:**
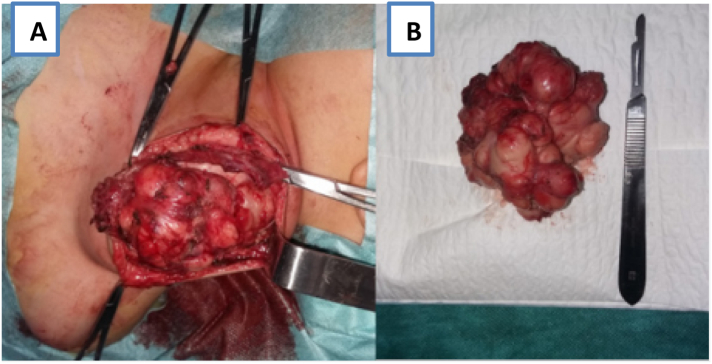
Per-operative image showing the supraclavicular mass being dissected (A) and operative piece (B).

The histological analysis of the biopsy was consistent with lipoblastoma. Surgical excision via a cervical approach was considered. Per-operatively, the lesion was well encapsulated fixed to the clavicle, in contact with the branches of the brachial plexus posteriorly. A complete surgical resection of the mass was performed, the subclavian vein was tightly adherent to the mass which was gently dissected, and the brachial plexus was seen and preserved (Fig. 3).

Histological examination showed an adipose lesion, divided into lobules by fibrous septa. The adipose tissue consisted of mature adipocytes of variable size mixed with single and multivacuolated lipoblasts. These findings were compatible with the initial diagnosis. The postoperative period was uneventful, one-year follow-up found no local recurrence.

### 2.2 Case 2

**Fig. 4:**
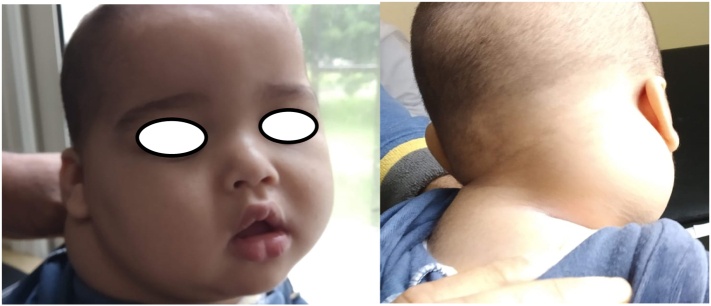
Pre-operative image showing an enlarging, painless right posterior neck mass.

A 8 month-old boy presented with a 4-month history of an enlarging, painless right posterior neck mass with no sign of compression, in a context of a conservation of general state. There was no history of trauma, infection or fever. Physical examination revealed a painless, non-pulsatile firm mass, measuring approximately 6 cm of large diameter, located in the right posterior triangle of the neck, starting at the midline and extending to the hairline superiorly and toward the base of the neck inferiorly (Fig. 4). with no inflammatory signs, no adenopathy or cranial nerves deficit were observed.

**Fig. 5:**
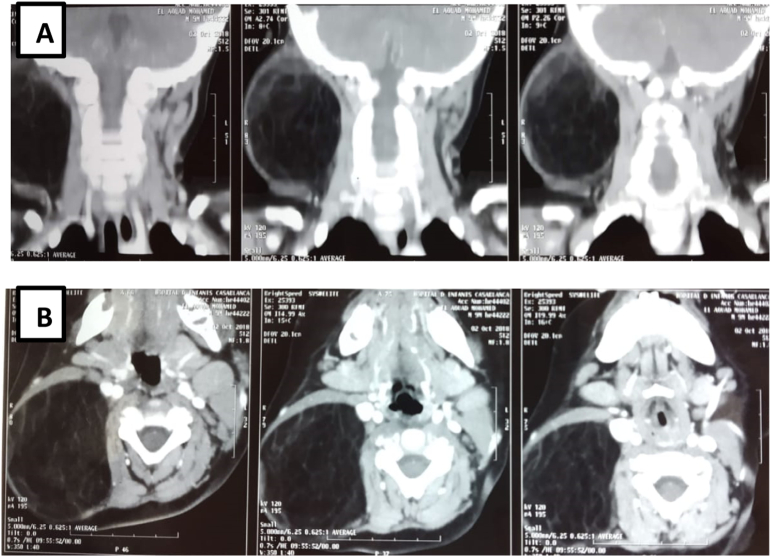
Neck CT scan, A/ coronal, B/ axial section: a well limited right latero cervical mass of a greasy density (61 × 50 × 65 mm) crossed by multiple septa.

Neck CT scan revealed a well limited right latero cervical mass of a greasy density, crossed by multiple septa, measuring 61 × 50 × 65 mm. The mass came into contact with the VJI which remains permeable. It pushes out the right sternocleidomastoid muscle and it pushes inwards the paravertebral muscles without sign of suffering (Fig. 5).

Surgical excision via a cervical approach demonstrated a well-encapsulated, soft, yellowish-white mass. The mass was successfully dissected without neurological compromise. Histological examination showed a lipoblastoma. The postoperative period was uneventful, 6 months follow-up found no local recurrence.

### 2.3 Case 3

**Fig. 6:**
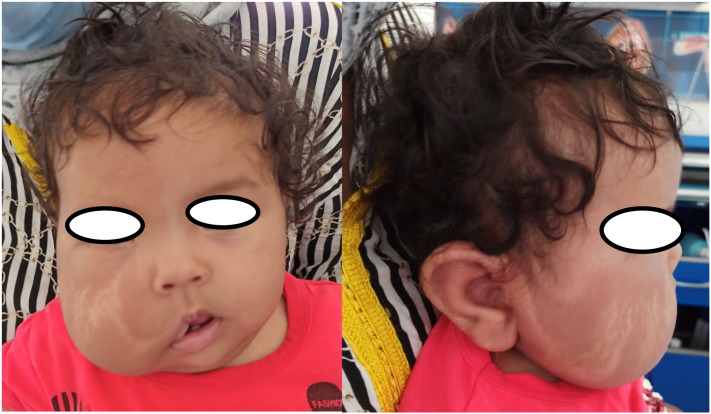
Right-sided facial mass causing noticeable facial deformity.

A 4-month-old girl, without a relevant history, presented with a painless right-sided facial mass growing progressively since birth. On physical examination, the mass was soft and nonpulsatile, located in the right cheek, overlying the mandible causing noticeable facial deformity and skin heterogeneous pigmentation (Fig. 6). The size of the mass was approximately 6 × 5 cm. Examination of the oral cavity was normal. No adenopathy or cranial nerves deficit was observed.

**Fig. 7:**
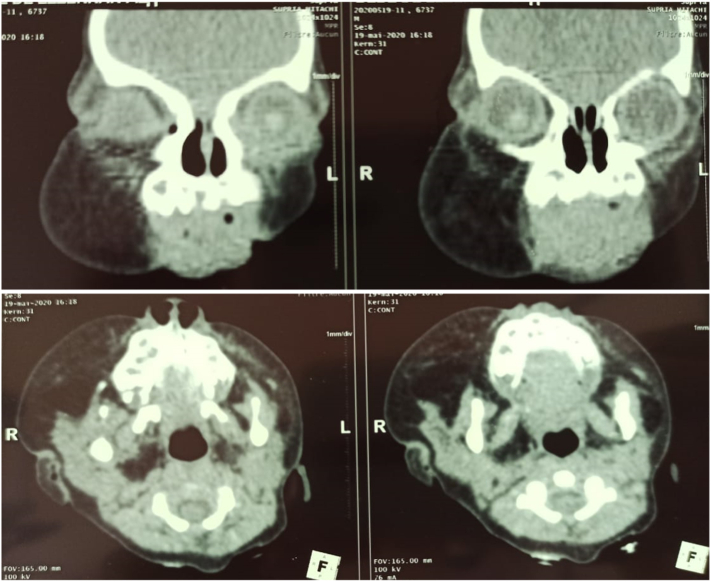
Axial and coronal sections of Facial CT scan showed a well-delimited homogeneous lesion measuring 54x50x28mm adjacent to the right mandibular cortex.

Facial CT scan showed a well-delimited homogeneous lesion measuring 54 × 50 × 28 mm adjacent to the right mandibular cortex without infiltration of the masseter (Fig. 7). The presumptive diagnosis was an adipose tissue tumor including initially lipoma, lipoblastoma and less likely liposarcoma.

The patient was not operated due to the high risk of facial paralysis and the mass did not increase in size after a 3-month follow-up.

## 3 Discussion

Lipoblastoma is a very rare benign tumor that originates from embryonic white fat cells. The majority of tumors occur in childhood and infancy, with 90% occurring before the age of 3 years old [10,11].

The tumor is most often located in the extremities and trunk, and occasionally in head and neck area (10–15%) [5]. Among this area, the neck is the most common location, but other locations may also be included such as the parotid gland, cheek, skin and orbit [12,13] It shows a male predominance and a rapid growth rate. In our study, the male to female ratio was 1/2.

The pathogenesis is unknown, though they are believed to arise from altered embryogenesis of human white fat and genetic predisposition, as chromosome 8 abnormalities may be implicated in the development of lipoblastoma [[15], [16], [17]].

Usually, it presents as a painless subcutaneous soft tissue mass growing progressively. Symptoms are related to the location and size or mass effect of the lesion. Its rapid growth may cause compressive symptoms caused by airway obstruction [13,18].

The presumptive diagnosis is performed by imaging: CT or MRI being more sensitive by showing the characteristics of the mass components. It shows the origin of the tumor, its composition and its anatomical extent, which helps in planning surgical resection [18]. After a thorough study of clinical and radiological data, the differential diagnosis should be limited to lesions showing similar characteristics such as lipoma, hibernoma, hemangioma and malignant lesions such as liposarcoma [[1], [2], [3],^10^,^24^,^14^].

The definitive diagnosis is made histologically, lipoblastoma shows lobulated appearance with mature, immature fatty cells and mesenchymal cells separated by septa [19]. The most important differential diagnosis of lipoblastoma is myxoid liposarcoma.

Since the distinction between the two entities cannot be performed on imaging. The age of the patient is significant. Liposarcomas are extremely rare in patients less than 10 years of age. Different lipomatous tumors tend to have characteristic chromosomal abnormalities. Lipoblastoma has been noted to consistently contain breakpoint abnormalities in chromosome 8q affecting PLAG1 when myxoid liposarcoma shows at (12;16) (q13;p11) translocation [20,21]. With a well-founded suspected diagnosis, treatment consists of complete resection of the tumor with free margins. If the entire tumor cannot be safely removed at the time of initial resection, a staged approach is recommended [22].

The prognosis is excellent, with a recurrence rate less than 25% being largely attributed to incomplete resection of large lesions or muscle infiltration. The evolution is unpredictable, spontaneous resolution and maturation into lipoma may occur [23].

## 4 Conclusion

Head and neck lipoblastoma are a rare benign tumor of embryonal fat cells occurring in infants and children, usually presenting as a progressive painless mass, rarely causing airway obstruction, nerve or vascular compression. Lipoblastoma should be suspected in case of heterogeneous fatty tumor, and included as a differential diagnosis of cervical masses in children younger than 3 years. Complete non-mutilating resection of the tumor is the treatment of choice. The prognosis is excellent despite large tumor size and local invasion. A regular follow-up is important for early detection of recurrences.

## Ethical approval

Written informed consent was obtained from the patient for publication of this case report and accompanying images. A copy of the written consent is available for review by the Editor-in-Chief of this journal on request.

## Funding

No funding was obtained for this study.

## Author contribution

Boutaina Merzouqi: acquisition of data, writing the article.

Mohammed Laachoubi: Corresponding author, writing the article.

Youssef Oukessou: study concept, writing the article.

Mohamed Mahtar: final approval.

## Guarantor

Mohammed Laachoubi.

## Registration of research studies

This is a case report that does not require a research registry.

## Declaration of competing interest

The authors of this article have no conflict or competing interests. All of the authors approved the final version of the manuscript.
